# COVID-19 Crisis: How to Avoid a ‘Lost Generation’

**DOI:** 10.1007/s10272-020-0908-y

**Published:** 2020-07-28

**Authors:** Dennis Tamesberger, Johann Bacher

**Affiliations:** 1 Wirtschafts-, Sozial- u. Gesellschaftspolitik, Kammer für Arbeiter und Angestellte, Volksgartenstraße 40, 4020 Linz, Austria; 2 Abtl. Empirische Sozialforschung, Johannes Kepler University, Inst. f. Soziologie, Altenbergerstr. 69, 4040 Linz, Austria

## Abstract

The spread of the coronavirus has made economic conditions difficult in many economic areas and has led to skyrocketing youth unemployment in most European countries. On the basis of simple model calculations, we estimate the consequences of the COVID-19 shutdown on youth unemployment in the European Union for the year 2020. According to our estimations, youth unemployment will increase from 2.8 to 4.8 million. The youth unemployment rate will increase to 26%, and the number of young people not in education, employment and training (NEET) will increase from 4.7 to 6.7 million. Policymakers at the national and international level should react as quickly as possible and make great efforts to avoid these negative scenarios. We suggest the introduction of a new European Youth Guarantee to ensure fiscal relief for those countries that suffer the most economically. It should be financed jointly by the EU and the respective member states. We suggest a new formula-based co-financing model in order to guarantee solidarity between the member states.

The shutdown of major parts of the economy to avoid the rapid spread of the coronavirus has led to skyrocketing unemployment rates in most countries. However, the labour market situation for young people has been especially difficult and this has rarely been recognised in public debates. The latest proposal from the European Commission (EC) has the promising title "Europe's Moment: Repair and Prepare for the Next Generation" and youth employment support is explicitly mentioned. The recovery plan has an impressive budget of €750 billion (EC, 2020a), which should leave room for the necessary focus on young people (EESC Workers' Group, 2020). On 1 July the EC presented the proposal "Youth Employment Support: a bridge to jobs for the next generation", with a suggested budget of €22 billion (EC, 2020c).

* Thanks are offered to Georg Feigl, Silvia Hofbauer, Nikolai Soukoup and Judith Vorbach for their helpful comments. For her academic assistance we are in debt to Denisa Husic.

**Dennis Tamesberger**, Chamber of Labour, Linz, Austria.

To give an idea of the extent of the problem, this paper estimates on the basis of simple model calculations the consequences of the current recession on youth unemployment and on the number of young people who are not in employment, education or training (NEET) in the European Union for the year 2020.

## Economic downturn and youth unemployment

Youth unemployment and general economic developments are highly correlated (Clark and Summers, 1982; Dietrich, 2013). Given a recessive economic situation, companies make employees redundant or hire fewer people due to a decline in orders. This affects young people disproportionately. On the basis of OECD data for the time period from 1970 to 2009, Bell and Blanchflower (2011) show that a 1% increase in the adult unemployment rate leads to a 1.79% increase in the youth unemployment rate. O'Higgins (1997) explains the high business cycle sensitivity of youth unemployment from a supply-side and demand-side perspective. From a supply-side point of view, young people tend to have a lower threshold when it comes to resigning or changing jobs as they have fewer firm-specific qualifications and fewer economic responsibilities. Even though these arguments seem plausible, the demand-side explanation has more weight: firms have lower opportunity costs if they make young employees redundant instead of older ones because they have invested less in their training, and young employees often have less protection against dismissal (last in, first out). Besides the aggregated demand with its major role in youth labour market outcomes, demo-

Table 1
**Forecast 2020 scenarios for the youth labour market in the European Union**

| Youth 15-24 years, EU27 | 2008 | 2009 | Change 2008-2009 Absolute | Change 2008-2009 Relative (%) | 2019 | 2020 forecast scenarios Optimistic IMF spring forcast | 2020 forecast scenarios Middle EC spring forecast | 2020 forecast scenarios Pessimistic Assumption |
|---|---|---|---|---|---|---|---|---|
| Real GDP growth rate, percentage change on previous year | | | | -4.30 | | -6.60 | -7.40 | -10.00 |
| Youth population in millions | 52.70 | 52.04 | -0.66 | -1.25 | 46.38 | 46.38 | 46.38 | 46.38 |
| Active labour force in millions[a] | 21.96 | 21.40 | -0.56 | -2.55 | 18.25 | 18.25 | 18.25 | 18.25 |
| Employed youth in millions | 18.44 | 17.04 | -1.40 | -7.59 | 15.49 | 13.69 | 13.47 | 12.76 |
| Unemployed youth in millions | 3.52 | 4.37 | 0.85 | 24.15 | 2.76 | 4.56 | 4.78 | 5.49 |
| Number of NEET individuals in millions[b] | 5.64 | 6.40 | 0.76 | 13.51 | 4.68 | 6.49 | 6.71 | 7.06 |
| Youth unemployment rate in % of labour force | 16.00 | 20.04 | 4.04 | 25.25 | 15.00 | 25.01 | 26.21 | 30.11 |
| Share of young people who are NEET (NEET rate in %) | 10.70 | 12.30 | 1.60 | 14.95 | 10.10 | 13.99 | 14.46 | 15.23 |

Notes: [a] Active labour force comprises unemployed (seeking employment) and employed. [b] Estimated via NEET rate in percent multiplied by youth population in million/100.

Source: Eurostat, lfsa_pganws, lfsa_pgaied, une_rt_a, edat_lfse_20, own calculations.

graphic developments and institutional factors play a part, such as employment protection legislation (e.g. Bassanini and Duval, 2006; Boeri et al., 2015), the vocational system (e.g. Breen, 2005; Cahuc and Hervelin, 2020; Quintini et al., 2007) and labour market policy (Tamesberger, 2015). Boeri et al. (2016) even point out that the rise in youth unemployment in Southern Europe during the Great Recession was in part an unintended consequence of pension reforms which increased the retirement age.

## Background of the forecast scenarios

During the Great Recession, specifically between 2008 and 2009, GDP in the EU27 countries decreased by 4.3% and the number of young people in employment decreased by 7.59%. The youth unemployment rate increased by four percentage points within one year to 20.04% in 2009. In relative terms, the increase amounted to 25% (see Table 1). On the basis of this experience, we derive our assumption that a decrease in GDP by 1% leads *ceteris paribus* to a decrease in youth employment of 1.77%, and the unemployment rate as well as the number of young people who are NEET will increase accordingly. In the current economic downturn, the International Monetary Fund (IMF) assumes for Europe a reduction in real GDP of 6.6% for the year 2020. The European Commission's spring forecast predicts an even deeper recession (-7.4% of real GDP) in the European Union. We use these forecasts as an optimistic and a middle scenario respectively in order to calculate the corresponding impact of the economic downturn on the youth labour market. Due to the considerable uncertainties in current forecasts, we add a third assessment, a pessimistic scenario, of -10% of real GDP for the EU27.

The youth unemployment rate for each member state was calculated as follows.[1] In the first step, the 2019 youth unemployment rate *YR* was computed as

$$YR_{i,\,2019} = \frac{Y_{i,\,2019}}{(Y_{i,\,2019} + X_{i,\,2019})}$$

for each country *i* in the sample, where $Y_{i,\,2019}$ is the absolute number of young unemployed people in country *i* and $X_{i,\,2019}$ is the absolute number of young employed people.

In a next step, the reduction in the number of employed young people was computed as

$$-\Delta X_i = GDP_i \cdot 1.765116 \cdot X_{i2019}$$

where $GDP_i$ is the predicted decrease of GDP for each country *i* by the EC spring forecast. For each country, we used the overall (EU27) elasticity of youth employment and economic

1 The sources used are the European Commission Spring 2020 Economic Forecast for $GDP_i$ and Eurostat for $Y_{i,2019}$ (yth_empl_090) and $X_{i,2019}$ (lfsa_pganws).

growth of 1.765116 during the financial and economic crisis in 2009. Of course, there is a variation in the elasticity among the member states. However, we assume that the Youth Guarantee implemented in the year 2013 had contributed to a certain convergence between the EU member states and therefore the average seems to be more realistic. This gives a first estimation that could be refined in a next step using country-specific elasticities and longer time series.

Finally, the number of employed young people $X_{i, 2020}$, the number of unemployed young people $Y_{i, 2020}$ and the youth unemployment rate $YR_{i, 2020}$ were estimated via the following formulae:

$$X_{i, 2020} = X_{i, 2019} - \Delta X_i,$$

$$Y_{i, 2020} = Y_{i, 2019} + \Delta X_i \text{ and}$$

$$YR_{i, 2020} = Y_{i, 2020}/(Y_{i, 2020} + X_{i, 2020})$$

All measures were computed for the age group 15 to 24 years.

### Results: The extent of the problem

If the predicted recession becomes a reality, the situation for young people will be dramatic. Youth unemployment will increase from 2.8 to 4.8 million (middle scenario in Table 1). The youth unemployment rate would increase to 26%, meaning that one in every four young people who wants to work will not find a job. Due to the limited relevance of the youth unemployment rate (Tamesberger, 2015), researchers focus on an additional indicator known as NEET (Maguire, 2013; Tamesberger et al., 2014). The underlying assumption is that the NEET status better captures young people who are at high risk of social exclusion. According to our estimation (see Table 1), the number of young people who are NEET will increase from 4.7 to 6.7 million, leading to a NEET rate of 14%, which means that one in every seven young people in the European Union will be in a NEET situation.

The optimistic scenario would be such that the youth unemployment rate in the EU27 would increase slightly above the 2013 level (see Figure 1). However, in the case of the middle or the pessimistic scenario, the youth unemployment rate would be the highest since the beginning of the recording on Eurostat. In all three scenarios, the NEET rate in the year 2020 will be historically high (see Figure 2).

Figure 3 provides country-specific estimations for the middle scenario. The expected youth unemployment rate in 2020 will vary between 16% and 46.3%. Greece, Spain and Italy have predicted values over 40%. Their youth unemployment rate was already high in 2019 and the EC spring forecast

Figure 1
**Development of youth unemployment rate, EU27**
in %

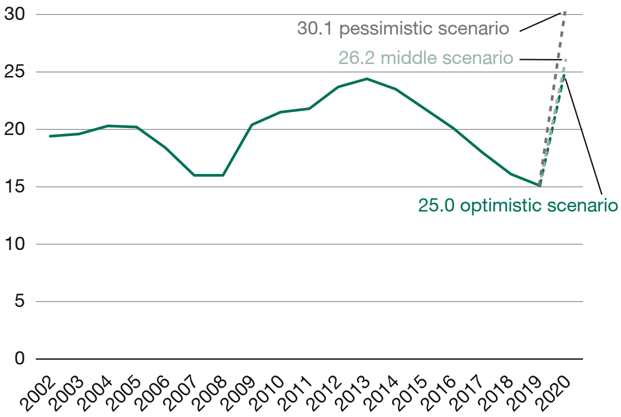

Source: Eurostat lfsa_urgan edat_lfse_20, own calculation.

predicts a reduction in real GDP of 9.4% to 9.7% for these countries (Greece: -9.7%, Spain: -9.4%; Italy: -9.5%). On the other hand, the Czech Republic, Germany and Poland are the countries with the lowest predicted youth unemployment rates. However, their values are equal or above 16% (Czech Republic: 16.0%, Germany: 16.6% and Poland: 16.7%).

### Negative consequences of youth unemployment

Unemployment generally, but especially during one's youth, has far-reaching consequences for individuals, society as a whole and economic development. Young adulthood is a sensitive period in life, characterised by socialisation and identity formation. Periods of unemployment during one's youth can have a negative impact later in life, which justifies the term 'lost generation' (Allegretto, 2013; Scarpetta et al., 2010). One month of unemployment at age 18-20 causes a permanent income loss of 2% (De Fraja et al., 2017). Morz and Savage (2006) show that a six-month unemployment period at the age of 22 leads to lower hourly wages of about 8% at the age of 23. At the age of 31, wages will be 3% lower in comparison to people without unemployment experience. With regards to health consequences, a vicious circle can be assumed: young people with health issues have a higher risk of becoming unemployed, and longer unemployment can in turn negatively influence health (e.g. Bacher et al., 2016; Bartley, 1994; Kuhn et al., 2009; McKee-Ryan et al., 2005; Schaufeli, 1997). A meta-analysis by Moser and Paul (2009) reveals both effects: the drift or rather selection effect of health problems on unemployment and the social causation effect of unemployment on health problems with reference to mental health. In addition, their study reveals both effects for school dropouts and university graduates alike. The negative consequences of youth

Figure 2
**Development of NEET rate, EU27**
in %

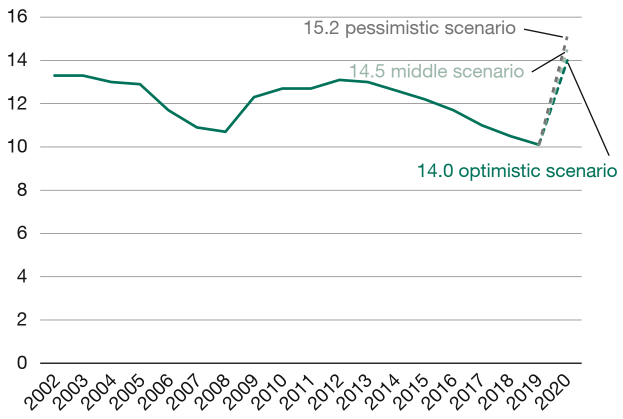

Source: Eurostat lfsa_urgan edat_lfse_20, own calculation.

Figure 3
**Estimated youth unemployment rate 2020 by country**
in %

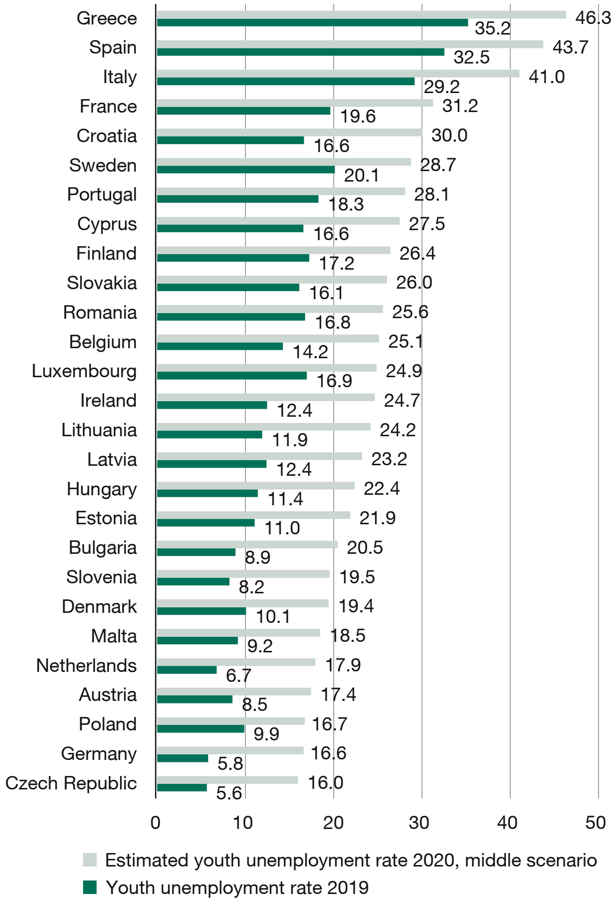

Source: Eurostat lfsa_urgan edat_lfse_20, own calculation.

unemployment on health can also be proven when the affected person turns 50 (Bell and Blanchflower, 2011).

In a qualitative study of long-term unemployed young people, Beelmann et al. (2001) identified three groups of social exclusion. Nearly half of them are characterised by high social exclusion in different fields of life (low opportunity to return to the labour market, precarious financial situation, social isolation, cultural and institutional exclusion). Eurofound (2015) calculated the economic costs of young people being NEET. They came to the conclusion that through the non-integration of young people during the Great Recession, European economies lost around €162 billion per year. Eurofound (2012) highlights that the consequences of youth unemployment are not merely economic but are also societal, with the risk of young people opting out of democratic and social participation in society. A decline of social capital (Putman, 2000) would be one of the consequences; an increase of extreme right-wing orientation and violence would be another (Heitmeyer, 1989). High youth unemployment in suburban areas may result in criminal or conflict subculture, as shown by Cloward and Ohlin (1960) and supported by recent studies (Body-Gendrot, 2013).

## A European Youth Guarantee as possible solution

In the face of skyrocketing youth unemployment during the Great Recession, the European Union introduced a Youth Guarantee scheme in 2013 to ensure that all young people under the age of 25 would receive a good-quality offer of employment, continued education, an apprenticeship or a traineeship within four months of becoming unemployed or leaving formal education (Council of the European Union, 2013). The Youth Employment Initiative was the main EU funding programme to roll out this Youth Guarantee with a total budget of €6.4 billion for the period from 2014 to 2020. It particularly supports regions where youth unemployment is higher than 25%. In 2015, the Commission proposed a 30% advance payment to eligible member states. Between 2014 and 2020, the Youth Guarantee will be partly financed for a total of €12.7 billion from the EU budget through the European Social Fund (ESF) and the Youth Employment Initiative (European Court of Auditors, 2015; Andor and Veselý, 2018). Even though the aim of the European Youth Guarantee was reasonable, its implementation has caused problems. Rautner et al. (2019) mainly criticise the insufficient funding total, the inadequate targeting as well as the quality of the programmes and the slow bureaucratic start. Also the European Court of Auditors (2015) highlighted the "adequacy of total funding" as one of the threats to the successful implementation of the Youth Guarantee.

### Sufficient funding

It would go beyond the scope of this article to propose a specific form of a redesigned European Youth Guarantee.

From our point of view, it would be necessary to introduce a new European Youth Guarantee promptly and with sufficient funding so as to ensure that those countries that suffer most economically are unburdened. The estimated necessary costs of a Youth Guarantee for the EU are around €45.4 billion per year. The background of this estimation is the experience of Sweden, where the national Youth Guarantee had very positive impacts at a relatively modest cost. In 2010, the estimated cost per participant of the Swedish Youth Guarantee plan was approximately €6,000 plus administrative costs (Escudero and Mourelo, 2015). Eurofound (2015) estimated it at €50.4 billion per year, which is still lower than the cost of not acting (around €162 billion per year).

If policymakers intend to provide adequate total funding, the majority must be financed by the EU budget with member states contributing to the Youth Guarantee according to their financial capacity. The latest proposal for a recovery fund from the European Commission includes this distribution idea as well with a budget of €750 billion titled "Europe's moment: Repair and Prepare for the Next Generation" (EC, 2020a), there should be room for a focus on young people (EESC Workers' Group, 2020). In addition, the EC (2020b) announced a programme called "Recovery Assistance for Cohesion and the Territories of Europe" (REACT-EU) that provides an additional fund of €55 billion from 2020 to 2022. Youth unemployment is mentioned as a distribution criterion. "The REACT-EU funding will be distributed among member states taking into account their relative prosperity and the extent of the effects of the current crisis on their economies and societies, including on youth unemployment" (EC, 2020b, 1). In our opinion, more money is immediately needed to successfully reduce youth unemployment. Therefore, we propose a new fund that should be endowed with €50 billion per year.

With reference to our prediction (middle scenario, see Table 2), the fund would guarantee that €10,400 is available on average for each unemployed young person. This amount is below the average cost of the non-integration of young people NEET (Eurofound, 2012, 2015).

Two-thirds of the fund should be financed by the EU and could be taken from the €750 billion recovery fund and from the REACT-EU programme if it is established in the foreseeable future. The member states should finance one-third according to their increase in youth unemployment.

### Fair distribution of EU funding

Our concept is inspired by the ideas of formula-based financing of educational systems (Levacic, 2008). Here, one main element of formula-based financing is to provide those schools with additional resources that have more disadvantaged students. More equity and transparency are important advantages of formula-based financing (Levacic, 2008). One main criticism is that the output is neglected (Levacic, 2008; Hanushek, 2003). Given the urgency, this argument does not carry the same weight. In the long term, formula-based financing can integrate the output (outcome) as an additional component.

In order to guarantee solidarity and to avoid a possible free-rider problem, the co-financing by a member state should decrease if the pandemic results in a higher increase in youth unemployment in that country than in others.

If the increase is zero, the EU will not co-finance. If the increase exceeds a certain threshold $t$, the EU will finance 100% of the Youth Guarantee. We can call this the solidarity threshold. A lower value symbolises more solidarity.

The contribution $c_i$ of a country to the fund is

$$c_i = n_i \cdot cf_i \cdot a_i$$

with $n_i$ being the number of unemployed young people for a certain reference date during the crisis (e.g. June 2020), $cf_i$ representing the co-financing factor (100% if there is no increase in youth unemployment and 0 if a certain threshold is passed), and $a_i$ being the normalising factor so that the sum $\sum c_i$ gives €16.5 billion.

If the increase in youth unemployment as a percentage for a country $i$ is denoted by $\Delta yi$ and the threshold by $t$, the co-financing factor is

$$cf_i = \begin{cases} 100 & \text{if } \Delta y_i \leq 0 \\ 100 - 100\,\dfrac{\Delta y_i}{t} & \text{if } \Delta y_i > 0 \text{ and } \Delta y_i \leq t \\ 0 & \text{if } \Delta y_i > t \end{cases}$$

The increase in youth unemployment is measured as the difference between the youth unemployment rate before the COVID-19 crisis and during or shortly after the crisis:

$$\Delta y_i = yr_{i,\,during} - yr_{i,\,before}$$

One possibility for normalising the threshold could assume that the country with the highest increase co-finances with a symbolic low percentage of $\pi$:

$$\pi = 100 - 100 \cdot \frac{\max(\Delta y_i)}{t} \Leftrightarrow t = \frac{100}{100 - \pi} \cdot \max(\Delta y_i)$$

If $\pi = 5\,\%$ the threshold is $t = \frac{100}{95} \cdot \max(\Delta_i)$

Table 2
**Parameters of the suggested new European Youth Guarantee**

| Parameters in thousands | Optimistic scenario | Middle scenario | Pessimistic scenario |
|---|---|---|---|
| Fund | €50,000 | €50,000 | €50,000 |
| Two-thirds financed by the EU. | €33,50 | €33,500 | €33,500 |
| One-third financed by countries according to the respective increase in youth unemployment. | €16,500 | €16,500 | €16,500 |
| Number of unemployed youth | 4,565 | 4,8 | 5,49 |
| Number of NEET individuals | 6,49 | 6,71 | 7,06 |
| Funding per unemployed youth | €11.0 | €10.4 | €9.1 |
| Funding per NEET youth | €7.7 | €7.5 | €7.1 |
| Cost of NEET (valorised values of Eurofound 2012) | €11.7 | €11.7 | €11.7 |

Source: Own calculation.

Of course, it is possible to lower the threshold and thus to demonstrate more solidarity. According to our calculation, the largest increase of the youth unemployment rate occurs for Croatia, with a value of 13.43. Hence, $t$ would have a value of 14.14.

After paying into the fund, each country receives an amount $s_i$ according to the number of unemployed young people $n_i$, the purchasing power parities (comparative price level) of the country $ppp_i$ and a normalisation factor $b_i$:

$$s_i = n_i \cdot ppp_i \cdot b_i$$

The normalisation factor guarantees that the sum $\sum s_i$ is €50 billion.

The specification of the parameters of the model requires further research. It might be useful to integrate additional social and economic factors as co-financing factors. Additional criteria might be the poverty rate as a measure of the social component and the general government gross debt as an economic component. Member states with a higher poverty rate and/or a higher debt rate should co-finance less because of their restricted opportunities. In this case, the co-financing factor contains three components (increase in youth unemployment, poverty rate, general government gross debt), which can be averaged by:

$$cf = w_{YR}\, cf_{YR} + w_{PR}\, cf_{PR} + w_{GGGD}\, cf_{GGGD}$$

where $cf_{YR}$ stands for the co-financing factor 'increase in youth unemployment', $cf_{PR}$ is the co-financing factor 'poverty rate', $cf_{GGGD}$ represents the co-financing factor 'general government gross debt' and $w_x$ stands for weight for co-financing factor x ($\sum w_x = 1$).

A general formula for calculating the co-financing factor is

$$cf_{x,i} = \begin{cases} 100 & \text{if } x \le x\min \\ 100 - 100\dfrac{x_i - x\min}{t_x - x\min} & \text{if } x > x\min \text{ and } x \le t \\ 0 & \text{if } x > t \end{cases}$$

where $w_i$ is the value for the country $i$ in co-financing factor x, $x\min$ is a defined minimum for co-financing factor x and $t_x$ is the solidarity threshold for co-financing factor x.

In the above example of youth unemployment, $x\min$ was set to zero.

## Conclusion

This paper sheds some light on the consequences of the COVID-19 shutdown on youth unemployment in the European Union. The presented estimations of youth unemployment on EU average and the country-specific estimations can serve as orientation for policymakers. However, the extent of the problem with a predicted increase of about two million young unemployed people is alarming. Because of the far-reaching negative consequences of youth unemployment, policymakers on national and international levels should act promptly and take great pains to avoid these negative scenarios.

As one possible solution to tackle increasing youth unemployment, we are suggesting a new European Youth Guarantee that should be endowed with €50 billion per year. The fund should be financed by the EU and the member states (two-thirds and one-third, respectively). We suggest a formula-based co-financing model in order to guarantee solidarity among the member states. The co-financing criteria could contain three components (increase in youth unemployment, poverty rate and general government gross debt). Further research on this topic would be worthwhile for several reasons. The expected high unemployment makes effective policy measures necessary. The proposed fund has the advantage of avoiding free-riding because each country has to pay in. On the other hand, it promotes solidarity. The formulisation objectifies the political discussion. Finally, the motivation of the member states is a given because each country receives more than it pays in.

This new European Youth Guarantee with a formula-based co-financing model would not only signal that the

European Union cares about the next generation, but also that it is keen to support economically and financially struggling regions. For those unemployed young people who have already been awarded a qualification, the Youth Guarantee should also serve as a job guarantee (Tcherneva, 2018), meaning that young people gain their first experience of employment in the public or the non-profit sector. This public job guarantee would benefit not only young people, but rather the society as a whole would benefit from socially and ecologically useful products or services. After all, the coronavirus pandemic has painfully proven just how essential public services are within the welfare state.
